# Impact of family bonding therapy on childhood screen addiction in urban Kerala

**DOI:** 10.6026/973206300221061

**Published:** 2026-02-28

**Authors:** KJ Shaijo, N Siva Subramanian, B Mahalakshmi

**Affiliations:** 1 Department of Paediatric Nursing, Nootan College of Nursing, Sankalchand Patel University, Visnagar, Gujarat, India; 2 Department of Psychiatric Nursing, Nootan College of Nursing, Sankalchand Patel University, Visnagar, Gujarat, India

**Keywords:** Family bonding therapy, screen addiction, parents, school-aged children, experimental study

## Abstract

Excessive screen use among parents and children has emerged as a growing behavioral concern affecting family relationships and child development. Therefore, it is of interest to evaluate the effectiveness of Family Bonding Therapy in reducing screen addiction among parents and children in an urban community of Kerala. Hence, a total of 136 parent-child pairs were allocated to experimental and control groups, with 68 pairs in each group. Post-test assessment was conducted at the end of the intervention period. Thus, we show a significant reduction in mean screen addiction scores among parents and children in the experimental group compared to the control group (p <0.001). No significant change was observed in the control group, highlighting the importance of family-centered behavioral interventions in addressing screen addiction.

## Background:

Excessive use of digital screen devices within families has emerged as a growing behavioral concern, affecting interpersonal relationships, emotional bonding and daily functioning [1]. Screen addiction is increasingly recognized as a modifiable behavioral condition influenced by environmental and relational factors rather than merely individual traits [2]. Family-based interventions have gained attention as effective strategies for addressing behavioral addictions by strengthening emotional connection, communication and shared activities within the family system [3]. Family Bonding Therapy emphasizes structured parent-child engagement, emotional responsiveness and shared quality time to promote healthier behavioral patterns [5]. Previous intervention-based studies have demonstrated that family-centered therapies are effective in reducing behavioral addictions, improving emotional regulation and enhancing family cohesion [6]. Interventions that encourage parents to actively participate in children's daily routines have been shown to reduce children's reliance on screens for stimulation and emotional comfort [7]. Moreover, strengthening parental involvement and supervision through therapeutic family activities has been associated with reduced problematic screen behaviors among both parents and children [8]. Structured family-based programs have been reported to improve parent-child communication, increase mutual engagement and reduce excessive screen exposure in home environments [10]. Therefore, it is of interest to evaluate the impact of Family Bonding Therapy in reducing parental and children's screen addiction in an urban community of Kerala.

## Methodology:

## Study design:

An experimental study with a pre-test and post-test control group design was adopted to evaluate the effectiveness of Family Bonding Therapy in reducing screen addiction among parents and school-aged children.

## Study setting and participants:

The study was conducted in selected urban residential communities of Cochin, Kerala. A total of 136 parent-child pairs were enrolled and allocated into experimental (n = 68) and control (n = 68) groups. Parents of children aged 6-12 years who regularly used screen devices were included.

## Intervention:

Family Bonding Therapy was administered to the experimental group for 30 days, which involved structured daily parent-child interaction activities for at least 30 minutes. The control group did not receive any intervention.

## Data collection tools:

Parental screen addiction was assessed using the Smartphone Addiction Scale (SAS) and children's screen addiction was assessed using the Problematic Media Use Measure-Short Form (PMUM-SF).

## Data analysis:

Data were analyzed using paired and independent t-tests to assess the effectiveness of the intervention.

## Results:

**Figure 1:**
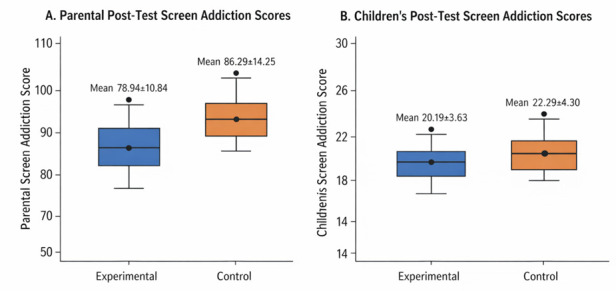
Box plot showing post-test parental and children's screen addiction scores in experimental and control groups (N=136).

Table 1 shows that parents in the experimental group had a significant reduction in screen addiction scores from pre-test to post-test (Mean difference = 9.87; t = 4.108; p < 0.001), whereas the control group showed no significant change (p = 0.597). Similarly, children in the experimental group demonstrated a significant reduction in screen addiction scores (Mean difference = 3.43; t = 4.317; p < 0.001), while changes in the control group were not significant (p = 0.086). Figure 1 visually supports these findings by showing lower median scores and reduced variability in post-test screen addiction scores among the experimental group compared to the control group for both parents and children, confirming the effectiveness of Family Bonding Therapy. Table 2 shows that post-test parental screen addiction had no significant association with selected demographic variables. Among children, a statistically significant association was observed only with type of family (χ^2^ = 12.219, p = 0.016), while all other demographic variables showed no significant association with post-test screen addiction levels.

## Discussion:

In the present study, Family Bonding Therapy produced a statistically significant reduction in both parental screen addiction (SAS) and children's screen addiction (PMUM-SF) in the experimental group, while the control group showed no significant change. These results support the concept that structured, daily parent-child engagement can modify household routines, reduce reliance on screens for stimulation/comfort and improve behavioral self-regulation within the family system. Wang *et al.* reported in a meta- analysis that family-based therapeutic approaches for internet addiction among adolescents and young adults demonstrate beneficial effects, supporting your finding that addiction-like digital behaviors are responsive to relational and family-centered treatment components [11]. Jones *et al.* further showed, through a large systematic review and meta-analysis, that screen-time interventions generally yield small but meaningful improvements and that goal-setting/planning- related behavior change techniques are linked with stronger effects-consistent with your structured daily activity approach [12]. Similarly, Marsh *et al.* highlighted that family involvement is a key ingredient in interventions aiming to reduce sedentary/screen behaviors among youth, aligning with the therapy's emphasis on shared routines and bonding [13]. Evidence syntheses focused specifically on children's screen-time reduction also support your outcomes. Wahi *et al.* found that interventions can reduce children's screen exposure overall, particularly when they include parent-focused components [14] and Maniccia *et al.* concluded that multicomponent programs targeting home routines are effective for reducing children's screen time and related behaviors [15]. Wu *et al.* also showed that RCT-based screen-time reduction interventions have measurable impact, reinforcing the plausibility of your observed pre-post reductions with a 30-day structured plan [16]. In preschool-focused evidence, Rico-González *et al.* summarized RCTs showing that family/community interventions can achieve meaningful reductions in screen time, which supports the feasibility of family-based approaches in community settings like urban Kerala [17].

Classic behavioral trials also strengthen the interpretation that changing home media routines can reduce excessive screen exposure. Robinson demonstrated that reducing children's television viewing through a structured approach can produce significant behavioral/health benefits [18] and Epstein *et al.* showed that reducing TV/computer use can improve outcomes related to children's weight and lifestyle patterns, indicating that screen reduction is achievable through deliberate household rule-setting and activity substitution [19]. Ni Mhurchu *et al.* provided evidence that device-based monitoring/limiting strategies can reduce television viewing, supporting the idea that practical home tools and structure can complement family bonding activities [20]. Your child results are also consistent with RCTs in younger ages: Yilmaz *et al.* demonstrated that a targeted preschool intervention can reduce screen time, supporting early-life behavioral shaping through structured engagement [21], while Birken *et al.* evaluated an office-based preschool screen-time reduction approach, reinforcing that structured guidance and parental participation are central mechanisms even when effect sizes vary [22]. In the Indian context, Poonia *et al.* showed that parental education can successfully limit early-childhood screen time, supporting the relevance of parent-mediated approaches in South Asian settings [23]. Sanders *et al.* reported feasibility and positive direction of change in a parenting-focused screen-time reduction study; supporting that parent training + routine change can be implemented at household level [4]. Finally, Qin-Xue Liu *et al.* demonstrated that multi-family group therapy can improve adolescent internet addiction outcomes, which conceptually supports your family bonding model as a relational mechanism for reducing digital dependence [9].

## Conclusion:

Family Bonding Therapy was found to be an effective intervention in reducing screen addiction among both parents and children in the experimental group. The significant improvement observed highlights the role of structured parent-child engagement in modifying daily routines and limiting excessive screen use. The absence of change in the control group strengthens the evidence for the intervention's effectiveness. Overall, the findings support the use of family-centered behavioral strategies to promote healthier screen habits and improve family well-being.

## Figures and Tables

**Table 1:** Effectiveness of family bonding therapy on parental and children's screen addiction (Pre-Post and Between Groups) (N=136)

**Outcome**	**Group**	**Pre-test Mean±SD**	**Post-test Mean±SD**	**Mean Change**	**Paired t (df=67)**	**p value**
Parental Screen Addiction (SAS)	Experimental (n=68)	88.81±17.11	78.94±10.84	9.87 ↓	4.108	<0.001***
	Control (n=68)	87.79±16.83	86.29±14.25	1.50 ↓	0.532	0.597 (NS)
Children's Screen Addiction (PMUM-SF)	Experimental (n=68)	23.62±5.47	20.19±3.63	3.43 ↓	4.317	<0.001***
	Control (n=68)	23.87±5.69	22.29±4.30	1.57 ↓	1.74	0.086 (NS)
NS = Not Significant;
** p<0.01; *** p<0.001.

**Table 2:** Association of post-test screen addiction level with selected demographic variables among parents and children (Merged) (N=136)

**Domain**	**Variable**	**χ^2^**	**df**	**p value**	**Result**
Parents (Post-test PSA level)	Age	2.065	4	0.724	NS
	Socio-economic status	0.034	2	0.983	NS
	Educational status	8.311	6	0.216	NS
	Working status	1.591	2	0.451	NS
	Type of family	2.229	4	0.694	NS
	Purpose of screen use	2.81	4	0.59	NS
Children (Post-test CSA level)	Age	5.125	4	0.275	NS
	Number of siblings	4.714	6	0.581	NS
	Number of family members	3.81	4	0.432	NS
	Socio-economic status	0.152	2	0.927	NS
	Educational status	3.741	4	0.442	NS
	Type of family	12.219	4	0.016	S*
	Working status of parents	0.518	2	0.772	NS
	Purpose of screen use	1.513	4	0.824	NS
	Access to screen devices	2.436	6	0.876	NS
NS = Not Significant;
S* = Significant at p<0.05.
